# Human Infection with Japanese Encephalitis Virus Genotype V, China, 2025

**DOI:** 10.3201/eid3210.251876

**Published:** 2026-10

**Authors:** Wenjing Zou, Yan Peng, Jing Li, Li Cai, Weifeng Tang, Junjing Yang, Xiang Li, Junqiang Xu, Guodong Liang

**Affiliations:** Hubei Province Center for Disease Control and Prevention, Wuhan, China (W. Zou, Y. Peng, J. Li, J. Yang, X. Li, J. Xu); Wuhan Center for Disease Control and Prevention, Wuhan (L. Cai, W. Tang); National Key Laboratory of Intelligent Tracking and Forecasting for Infectious Diseases, National Institute for Viral Disease Control and Prevention, Chinese Center for Disease Control and Prevention, Beijing, China (G. Liang)

**Keywords:** Japanese encephalitis virus, genotype 5, meningitis/encephalitis, vector-borne, vector-borne infections, mosquito-borne, zoonoses, viruses, viral infection, China

## Abstract

Japanese encephalitis virus genotype V (JEV GV) has caused multiple cases of viral encephalitis in South Korea. We report a human case of JEV GV in China, confirmed by serology, sequencing, and quantitative reverse transcription PCR. We confirm JEV GV infection in China, highlighting the persistent threat of this virus.

Japanese encephalitis virus genotype V (JEV GV) was initially identified in 1952 from viral encephalitis patients in Malaysia and Singapore (*1*). JEV GV was detected again after an ≈60-year hiatus when it was isolated from field-collected *Culex tritaeniorhynchus* mosquitoes in Tibet (Medog County), China, in 2009 (*2*). Subsequently, JEV GV has been associated with multiple cases of viral encephalitis in South Korea and is now recognized as an emerging pathogen with considerable public health significance (*3*). Despite its reemergence, surveillance data from mainland China after 2009 had detected exclusively JEV genotype I (GI) in both mosquito populations and human clinical samples, suggesting a possible discontinuity in the circulation of JEV GV in this region (*4*). We report a fatal case of JEV GV infection, highlighting the persistent and evolving threat posed by this genotype.

A male neonate was delivered vaginally on October 6, 2025. Both mother and neonate were in good health at discharge. Neither had a recent history of travel to or residence in a JEV-endemic area outside of Wuhan, China. Laboratory testing in the mother indicated no evidence of recent or past JEV infection (IgM- and IgG-negative and JEV quantitative reverse transcription PCR [qRT-PCR]–negative 3 days after illness onset in infant) and she had not been vaccinated against JE. On day 21 after birth, a high fever (39.3°C) developed, and the neonate was admitted to Wuhan Children’s Hospital (Wuhan, China). During hospitalization (October 27–November 6, 2025), clinical manifestations were characterized by persistent high fever (37.8–40.0°C), coma, and convulsions. A diagnosis of suspected viral encephalitis was made, and the neonate unfortunately died on November 7, 2025.

We collected 5 specimens for laboratory testing: 2 cerebrospinal fluid (CSF) samples and 3 serum samples. The initial CSF sample, taken at admission (October 27, 2025), tested positive for JEV nucleic acid by qRT-PCR. Laboratory testing detected JEV-specific IgM in the serum sample collected on day 10 of illness. All other specimens were negative for both JEV RNA and IgM (Figure 1) (*5*).

**Figure 1:**
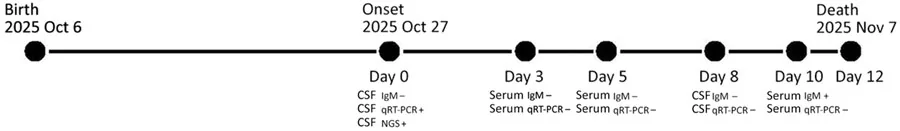
Timeline of a human case of Japanese encephalitis virus genotype V infection in China, 2025. Timeline spans from birth to death, indicating the timing of specimen collection, specimen types, and corresponding laboratory results. CSF, cerebrospinal fluid; IgM, Japanese encephalitis virus IgM detected by capture ELISA; NGS, next-generation sequencing; qRT-PCR, quantitative reverse transcription PCR; +, positive; –, negative.

We conducted next-generation sequencing on the CSF sample collected at admission (*6*). We assembled and designated the complete genome of JEV as the neonate strain (GenBank accession no. PZ393248). Phylogenetic analysis on the basis of the open reading frame sequences of JEV genotypes I–V (GI–GV) revealed that GV segregated into 2 distinct clades. One clade exclusively contained the 1952 prototype strains (Muar from Malaysia and Tengah from Singapore), whereas the other comprised all contemporary isolates, including the neonate strain, the XZ0934 strain (Tibet, China, 2009), and viruses obtained from human cases and mosquitoes in South Korea since 2015 (Figure 2, panel A). The positive qRT-PCR result for JEV in the admission CSF sample, together with the sequencing data, confirmed that the viral encephalitis in this neonate was caused by JEV GV. Further phylogenetic analysis of JEV GV envelope gene sequences revealed that all GV strains fell into 3 distinct evolutionary clades: clade A (the 1952 prototype strains), clade B (including the neonate and XZ0934 strains), and clade C (comprising all South Korea isolates) (Figure 2, panel B). Those results demonstrated a clear phylogeographic pattern of JEV GV, reflecting independent evolution of distinct viral populations in mainland China and South Korea after the reemergence of JEV GV in 2009.

**Figure 2:**
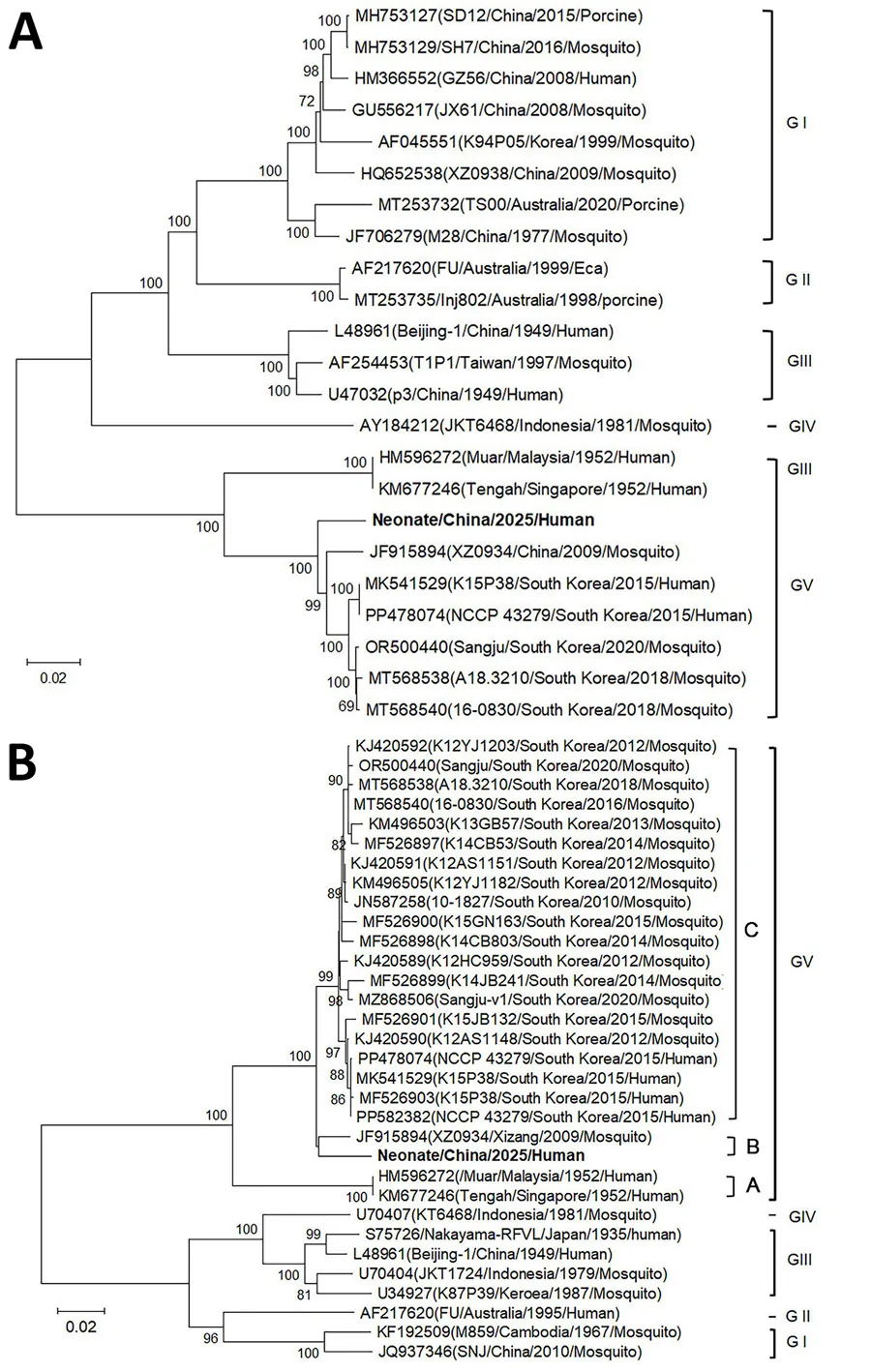
Phylogenetic trees of Japanese encephalitis virus (JEV) genes recovered from a human case in China, 2025. A) Phylogenetic tree built on the basis of the nucleotide sequences of the open reading frame of JEV. B) Phylogenetic tree built on the basis of the nucleotide sequences of the envelope gene of JEV. We constructed the phylogenetic trees by using the neighbor-joining method in MEGA 12.0 software (https://www.megasoftware.net). Neonate sequence from this study is in boldface. Branch numbers indicate percent bootstrap support values. Scale bars indicate nucleotide substitutions per site.

JEV GV has been consistently detected in multiple mosquito species in South Korea, where it has replaced GI as the dominant genotype, and human cases have been reported since 2015 (*2*,*7*). In contrast, since the initial isolation of JEV GV from mosquitoes in Tibet, China, no human cases of JEV GV had been reported in mainland China, nor was it reisolated from vector populations. However, a retrospective serologic analysis of laboratory-confirmed JE cases from 2018–2020 identified 2 patients with possible JEV GV infection in eastern China (Shandong and Zhejiang provinces) (*8*). During previous surveillance in Wuhan in 2009–2010, JEV GI was isolated from mosquitoes, whereas GIII had been detected in earlier mosquito collections (*9*). Together with the identification of the neonate strain in Central China and the earlier detection of the XZ0934 strain in Tibet, our findings confirm the established presence of JEV GV in southwestern, central, and eastern China. Of note, in recent years, JE cases caused by JEV GIV and GV have been increasing, with geographic expansion into Australia and South Korea (*3*,*10*). The detection of this JEV GV case in mainland China, together with those trends, emphasizes the urgent need to strengthen genotype-based detection and surveillance of JEV for effective Japanese encephalitis prevention and control.
